# Preclinical evaluation of 3D-Printed orthodontic aligners using an electro-typodont model

**DOI:** 10.3389/fbioe.2025.1650447

**Published:** 2025-11-10

**Authors:** Ammar A. Al Shalabi, Shaima Malik, Hoon Kim, Abdulaziz Alhotan, Ahmed Ghoneima, Tarek M. Elshazly

**Affiliations:** 1 Department of Orthodontics and Pediatric Dentistry, Hamdan Bin Mohammed College of Dental Medicine (HBMCDM), Mohammed Bin Rashid University of Medicine and Health Sciences (MBRU), Dubai, United Arab Emirates; 2 Division of Orthodontics and Dentofacial Orthopedics, Eastman Institute for Oral Health, University of Rochester Eastman Institute for Oral Health, Rochester, NY, United States; 3 Research Institute of Agriculture and Life Sciences, College of Agriculture and Life Sciences, Seoul National University, Seoul, Republic of Korea; 4 Department of Dental Health, College of Applied Medical Sciences, King Saud University, Riyadh, Saudi Arabia; 5 Section of Orthodontics, School of Dentistry, University of California Los Angeles (UCLA), Los Angeles, CA, United States; 6 Oral Technology, Dental School, University Hospital Bonn, Bonn, Germany

**Keywords:** biomechanics, orthodontics, dental models, orthodontic appliances, printing

## Abstract

**Objectives:**

The use of 3D printing in orthodontic aligner production addresses several limitations of conventional thermoforming. However, existing experimental techniques for evaluating aligner efficacy remain restricted. This study aims to introduce a novel experimental approach employing an electric typodont model to assess the effectiveness of 3D-printed orthodontic aligners in correcting rotation of the maxillary right central incisor (Tooth 11).

**Materials and Methods:**

An electric typodont, equipped with heat-activated wax blocks, simulated four rotational severities of Tooth 11: 22°, 32°, 42°, and 52°. Digital scans were processed in Maestro 3D software to design virtual treatment plans, from which four sequential aligners were fabricated per severity level. In total, 240 aligners were 3D-printed, using Tera Harz TC-85 resin, in three thicknesses: 0.50, 0.75, and 1.00 mm. Each aligner underwent a 10-minute heating cycle, followed by a 10-min cooling period. Tooth rotation was measured manually using a protractor relative to a fixed baseline. The procedure was repeated five times per subgroup, with repositioning guided by custom guiding stents.

**Results:**

Across all aligner thicknesses, 80.0%–93.1% of the planned rotational correction was achieved by the fourth aligner, leaving residual rotations of approximately 4°–5°. Higher initial rotations resulted in a greater percentage of corrections (p < 0.001). The 0.50-mm and 1.00-mm aligners demonstrated faster early-stage correction, whereas the 0.75-mm aligner exhibited a more gradual and consistent derotation pattern throughout the treatment stages (p < 0.001).

**Conclusion:**

The electric typodont appears to be a reliable pre-clinical tool for evaluating the effectiveness of aligners. Furthermore, 3D-printed aligners successfully achieved incisor derotation without the use of attachments. Furthermore, while variations in aligner thickness influenced the dynamics of derotation, they did not alter the ultimate correction outcome.

## Introduction

Clear aligners are personalized, transparent plastic appliances primarily designed to progressively correct mild to moderate dental misalignments, although they may also be applied in more complex cases when combined with auxiliaries (Tamer et al., 2019). Given the inherent stiffness of the plastic material, each aligner can induce only minimal positional changes in the teeth. Consequently, complete treatment requires a sequence of aligners, with each one programmed to implement small, incremental adjustments (Upadhyay and Arqub, 2022). This stepwise approach can become resource-intensive, especially in complex cases, due to the high number of aligners required and the associated material consumption (Elshazly et al., 2022a).

Recent advancements in intraoral scanning, 3D printing, and computer-aided design/computer-aided manufacturing (CAD/CAM) technologies have significantly enhanced the precision and customization of aligner fabrication (Moutawakil, 2021). Continuous innovative efforts aim to streamline treatment protocols, reduce treatment duration and costs, and improve clinical outcomes (Atta et al., 2023). Despite advancements in aligner biomechanics, discrepancies often persist between planned and clinically achieved tooth movements (Ayidağar and Kamiloğlu, 2021; Papageorgiou et al., 2020). Furthermore, there is no commonly accepted aligner geometry capable of optimizing the device for various types of tooth movement. This underscores the need for further research to strengthen the evidence base for achieving predictable tooth movements with aligners (Elkholy et al., 2023).

Numerous variables have been shown to influence treatment accuracy, including activation and staging of aligner steps (Min et al., 2010; Li et al., 2016; Jedliński et al., 2023), aligner’s thickness (Liu and Chen, 2015), as well as edge extension and trimming design of the aligners (Gao and Wichelhaus, 2017; Brown, 2021; Elshazly et al., 2022b; Elshazly et al., 2023a). Moreover, the mechanical behavior and clinical performance of aligners are largely governed by the properties of the materials used in their fabrication (Cremonini et al., 2022; Momtaz, 2016). Conventional thermoformed aligners are typically fabricated from single-layer polymers such as PETG or TPU, though multi-layer hybrids have been introduced to improve mechanical strength and comfort (Elshazly et al., 2024a). More recently, shape memory polymers (SMPs) have emerged as a novel material capable of reducing the number of aligners needed by recovering their original shape under specific stimuli, thereby applying sustained forces over larger tooth movements (Atta et al., 2023).

Traditional thermoforming techniques have been reported to degrade the mechanical integrity of aligner materials due to thermal and physical deformation during processing (Golkhani et al., 2022; Dalaie et al., 2021). In response, 3D printing has emerged as a promising alternative, offering greater precision, better control over geometric features and thickness (Elshazly et al., 2022b; Koenig et al., 2022), reduced material waste, and lower production costs (Peeters et al., 2019).

To better understand the forces and mechanics involved in aligner therapy, researchers have applied a variety of numerical and experimental approaches. These include finite element modeling (Elshazly et al., 2023b), direct force measurements using integrated sensors (Xiang et al., 2021), pressure-sensitive films (Elshazly et al., 2024a), and customized biomechanical devices (Elkholy et al., 2017). Other methods employed for assessing aligner performance include photoelastic stress analysis (Hamanaka and Nakamura, 2016), full-field digital image correlation for strain measurement (Maia and França, 2020), and optical tracking systems applied to typodont models (Li and Wang, 2018). Typodonts, in particular, serve as useful sensor-free, pre-clinical models for evaluating the mechanical effects of sequential aligners (Elshazly et al., 2021).

The current study introduced a new method utilizing an electrically controlled typodont to evaluate the performance of 3D-printed aligners in achieving controlled tooth movements. This advanced electric typodont offers several practical advantages for orthodontic simulation. Unlike traditional models, it eliminates the need for hot water baths and allows real-time visualization of tooth movement. Moreover, electrically controlled heating begins at the root level, simulating natural tooth displacement more accurately. Furthermore, its wax system mimics anatomical structures by using harder wax for cortical bone and softer wax for spongiosa. This clean, quick, and user-friendly device is ideal for experimental research purposes and provides flexible setup options, making it efficient to operate (Ghoneima and Al Ali, 2024). Different degrees of rotation of the maxillary central incisor were evaluated using 3D-printed aligners of varying thicknesses. The null hypothesis stated that no significant differences would be found between the groups.

## Materials and methods

**FIGURE 1:**
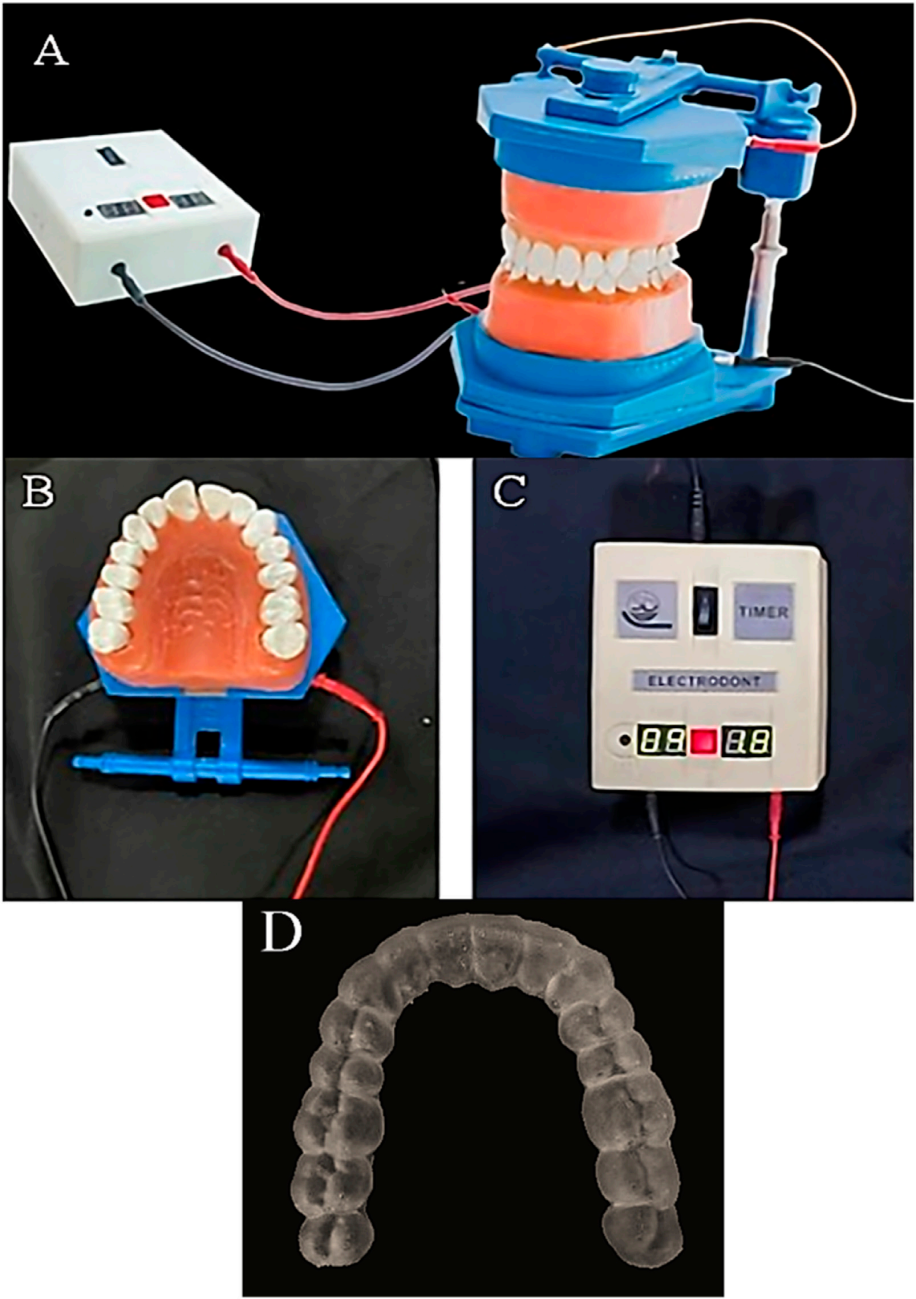
Components of the electric typodont system used in the study. **(A)** Full view of the electric typodont (ElectroDont). **(B)** Close-up of the upper dental arch of the ElectroDont. **(C)** Timer and power supply controlling the typodont’s movements. **(D)** Guiding positioning stent used to standardize tooth positioning and aligner placement.

An electrically operated typodont (Electro-Dont; Savaria-Dent, Budapest, Hungary) (Figure 1) was utilized to simulate controlled rotational movements of the upper right central incisor (Tooth 11) in this study. Unlike conventional typodonts—where teeth are typically fixed in place or manually repositioned—this advanced device allows for precise, repeatable tooth movement through a programmable heating system. The Electro-Dont consists of complete maxillary and mandibular dental arches with acrylic teeth embedded in a specialized wax matrix. Each individual tooth is surrounded by an electric coil connected to an external control unit with a programmable timer. When activated, the circuit delivers electrical energy to the coils, generating heat that gradually softens the wax around the target tooth. This process mimics the biological mobility of teeth, allowing predefined movements—such as rotation, tipping, or translation—to occur in a controlled and standardized manner. Following the heating phase, the system automatically initiates a cooling cycle of equal duration. As the wax re-solidifies, the tooth is stabilized in its new position without manual interference, ensuring accurate and reproducible outcomes. In contrast to conventional typodonts, which often require mechanical manipulation or physical resetting between simulations (Elshazly et al., 2021), the Electro-Dont offers a hands-free, programmable, and repeatable method for studying complex tooth movements under standardized conditions^1^. This makes it particularly advantageous for preclinical evaluations of orthodontic appliances, such as clear aligners, where precision and consistency are essential.

The typodont was digitized using an iTero intraoral scanner (Align Technology, San Jose, CA, United States) to generate a virtual model in STL (Standard Tessellation Language) format. The STL file was imported into Maestro 3D Ortho Studio software (AGE Solutions, Pontedera, Italy) for digital treatment planning and virtual tooth setup. Within the software, rotation, tip, torque, and center of rotation can be precisely adjusted according to the planned tooth movement, after which the clear aligner is digitally designed.

**FIGURE 2:**
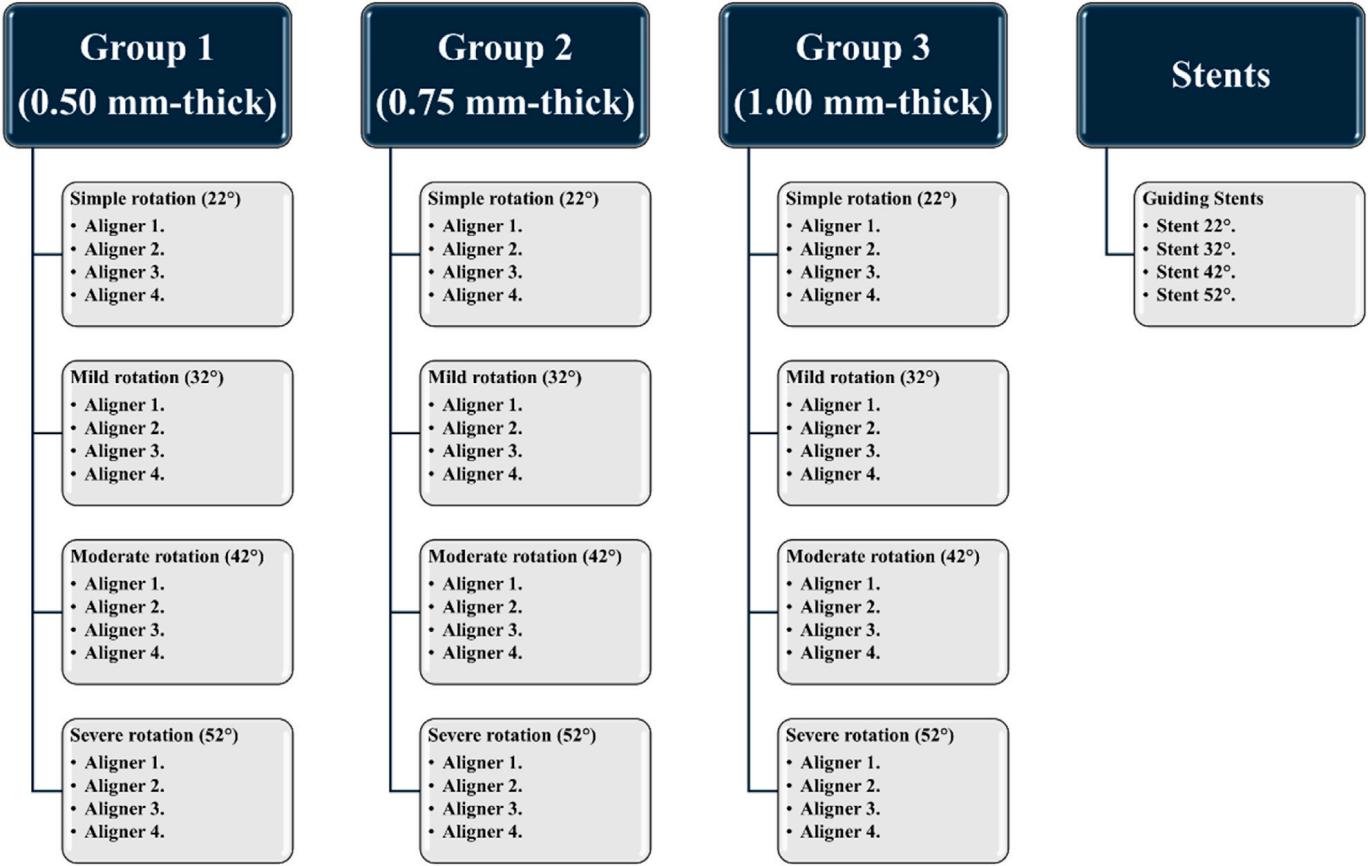
A scheme showing the classification of the current study groups, based on aligner thickness and degree of rotation of the upper central incisor.

**FIGURE 3:**
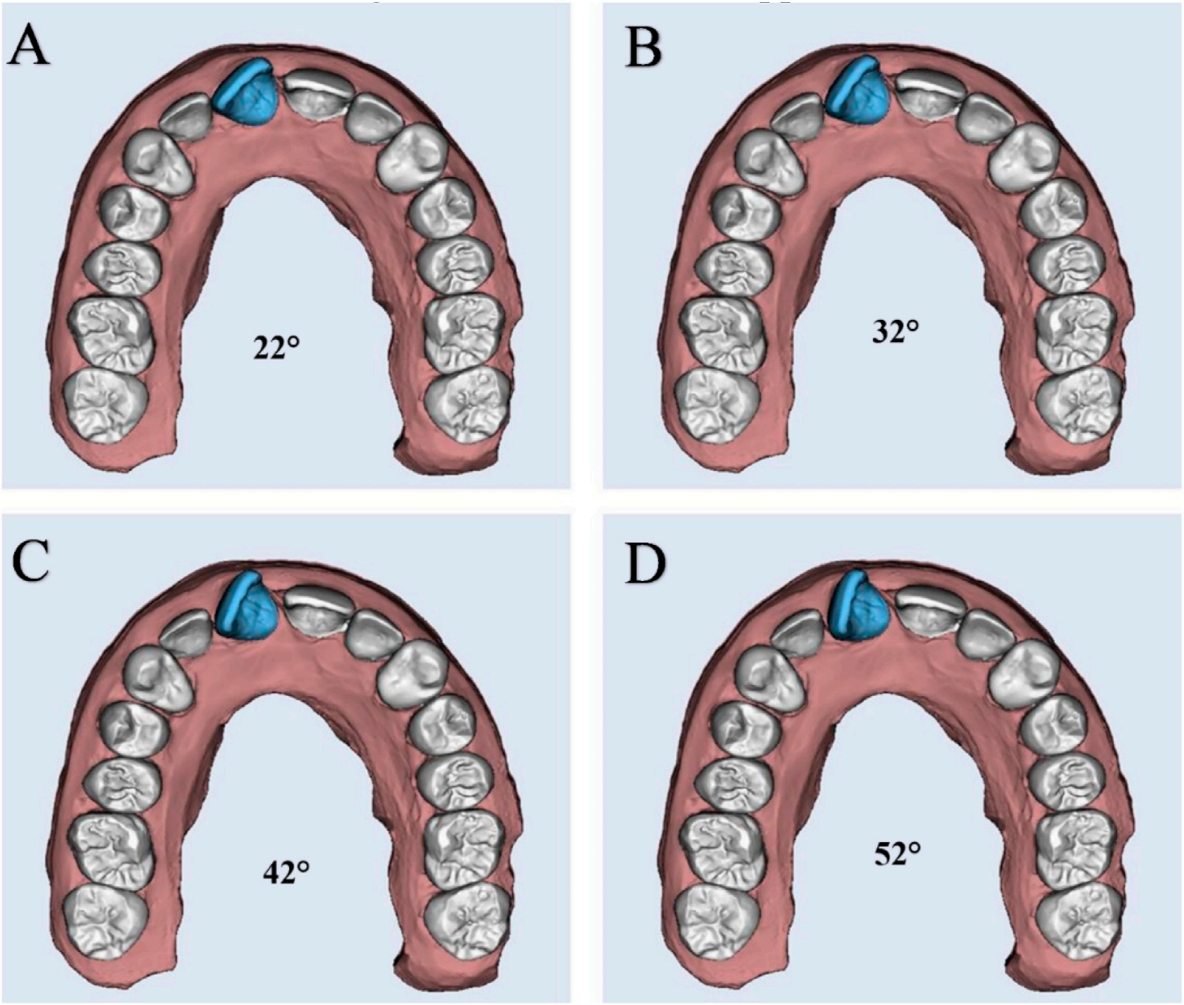
The digitally planned degrees of rotation of maxillary right central incisor selected in this study: **(A)** Simple rotation: 22°; **(B)** Mild rotation: 32°; **(C)** Moderate rotation: 42°; **(D)** Severe rotation: 52°.

In total, 240 clear aligners were fabricated using 3D-printing technology. These were categorized into three primary groups (n = 80 per group) based on material thickness: Group 1 (0.50 mm), Group 2 (0.75 mm), and Group 3 (1.00 mm). Each group was further subdivided into four subgroups (n = 20 per subgroup; n = 5 per test sample) according to the degree of planned rotational correction for Tooth 11 (Figures 2, 3):

- Subgroup A: simple rotation (22°)
- Subgroup B: mild rotation (32°)
- Subgroup C: moderate rotation (42°)
- Subgroup D: severe rotation (52°)

For each subgroup, a series of four sequential aligners was designed (Figure 2). The total planned correction for each initial rotation severity was evenly distributed across the four aligners (Aligners 1 through 4), with each aligner programmed to correct approximately 25% of the initial rotation. Accordingly, this corresponded to ∼5.5° per aligner in the 22° group, ∼8° per aligner in the 32° group, ∼10.5° per aligner in the 42° group, and ∼13° per aligner in the 52° group. By the end of the fourth aligner, the full correction of the respective rotational displacement was expected.

To initiate each test condition consistently, four rigid positioning stents were also fabricated from a photopolymer resin (Grey Resin 1 L; Formlabs, Somerville, MA, United States) (Figure 1). These guiding stents were used to preset the initial rotated position of Tooth 11 before the start of the testing cycle.

**FIGURE 4:**
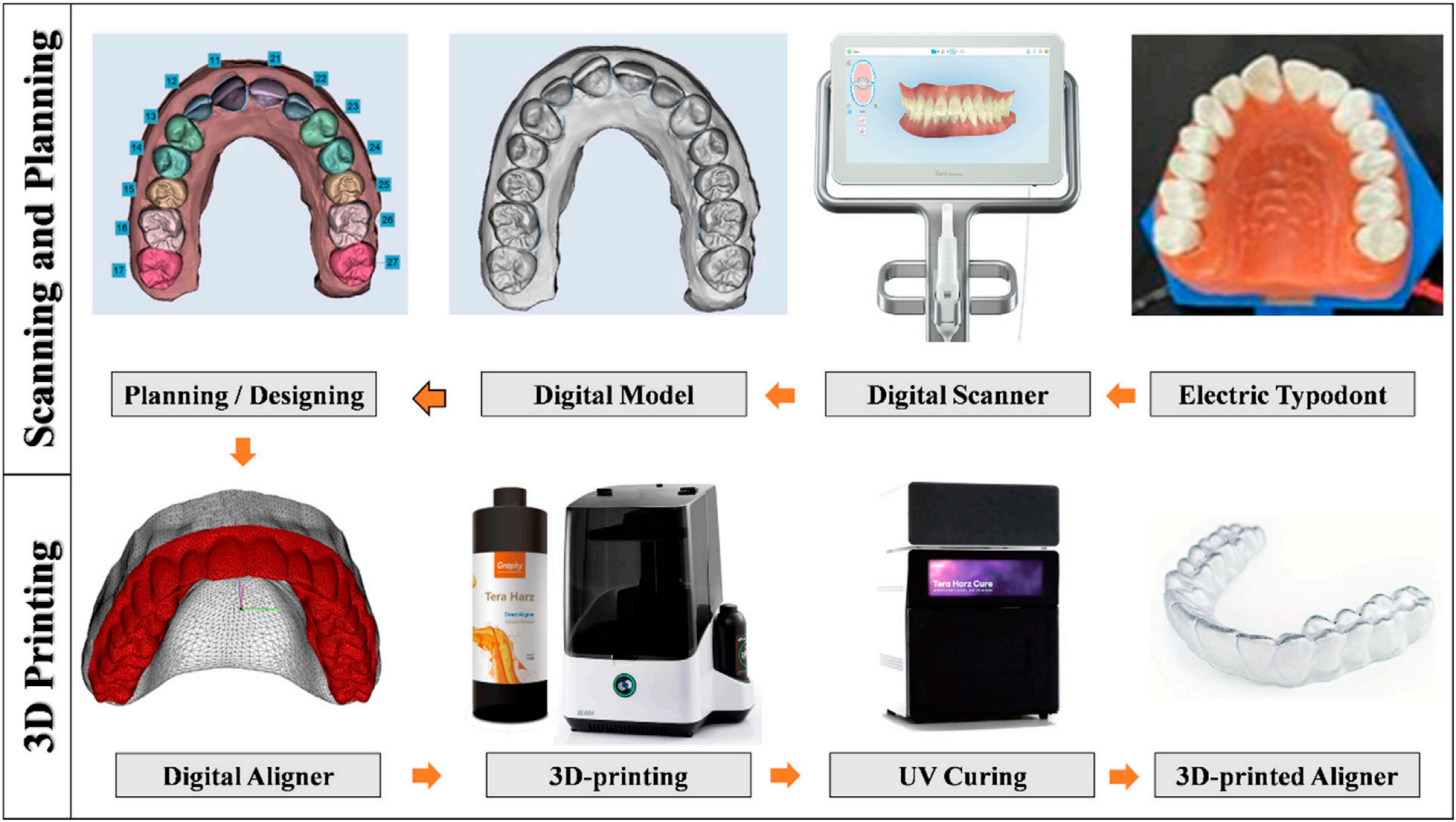
A schematic showing the workflow of the scanning process, treatment planning, aligner designing, and 3D-printing of the aligner in the current study.

All aligners were manufactured using Tera Harz TC-85 resin^**^ (Graphy, Seoul, South Korea) and produced with a DLP-based 3D printer (Uniz NBEE; Uniz, CA, United States), utilizing a 100 µm layer resolution and incorporating a straight trimming line extended by 2 mm. Post-printing, the aligners underwent ultraviolet (UV) light curing at a wavelength of 405 nm for 25 min under a nitrogen atmosphere, following the manufacturer’s specified protocol, using the Tera Harz Cure system (Graphy, Seoul, South Korea) (Figure 4).

Following the placement of the aligner onto the typodont, the ElectroDont system was activated to initiate a thermal cycle consisting of 10 min of controlled heating, immediately followed by a 10-min cooling phase. To ensure complete stabilization, the typodont was then submerged in room temperature water for an additional 2 min. After cooling, the aligner was gently removed to prevent any unintended tooth displacement, and the ElectroDont was scanned to record the resulting tooth position. At the end of each test cycle, the rotation of Tooth 11 was adjusted to its initial test position using the appropriate guiding stent.

**FIGURE 5:**
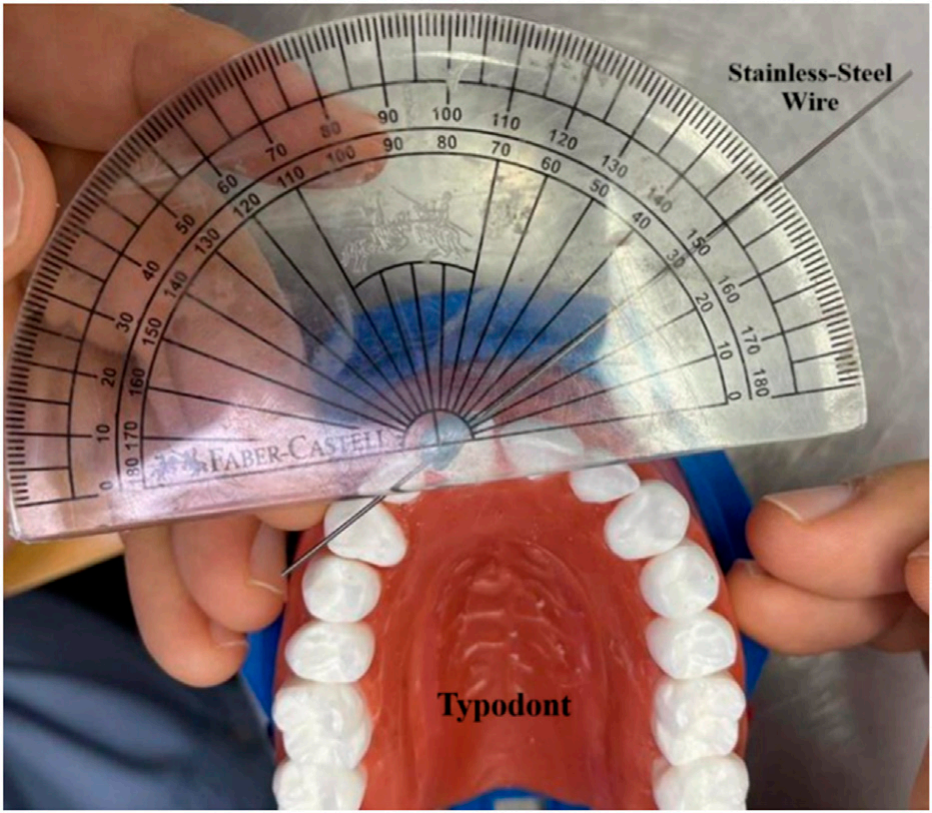
Manual measurement of the degree of rotation of Tooth 11 using a protractor. Rotation was quantified by attaching a wire along the incisal edge of Tooth 11 and measuring the angle between it and a baseline drawn to the mesial marginal ridge of Tooth 21.

To quantify the degree of rotation, a straight stainless-steel wire was affixed tangentially along the incisal edge of the rotated Tooth 11 using a small amount of wax for stabilization. A baseline reference was created by drawing a line from the midpoint of the incisal edge of Tooth 11 to the mesial marginal ridge of the adjacent Tooth 21, which had been previously marked. The angle formed between the wire and this baseline was measured using a protractor (Figure 5). This measurement protocol was consistently applied across all aligners and test conditions. For reliability assessment, the entire experimental procedure was repeated 5 times across all subgroups (n = 5). Moreover, the percentage correction of rotation was calculated for each group using the formula:

$$\%\;Correction=\frac{\left(Planned\;Rotation-Final\;Achieved\;Rotation\right)}{Planned\;Rotation}\times 100.$$

### Statistical analysis

*A priori* calculations showed that with 60 samples (n = 20 per thickness group), the study had 80% power to detect medium-to-large differences between aligner thicknesses (Cohen’s f ≈ 0.41) at α = 0.05. For the change from Aligner 1 to Aligner 4, the sample size provided over 80% power to detect large within-subject effects (Cohen’s dz ≥ 0.75).

Data were analyzed using SPSS V.28.0 (SPSS, Chicago, IL, United States). Results are reported as mean ± standard deviation. Normality was tested with Shapiro–Wilk. Paired t-tests were used for two related groups, and repeated-measures ANOVA with LSD post-hoc testing was applied for multiple measurements. A p-value <0.05 was considered statistically significant.

## Results

**FIGURE 6:**
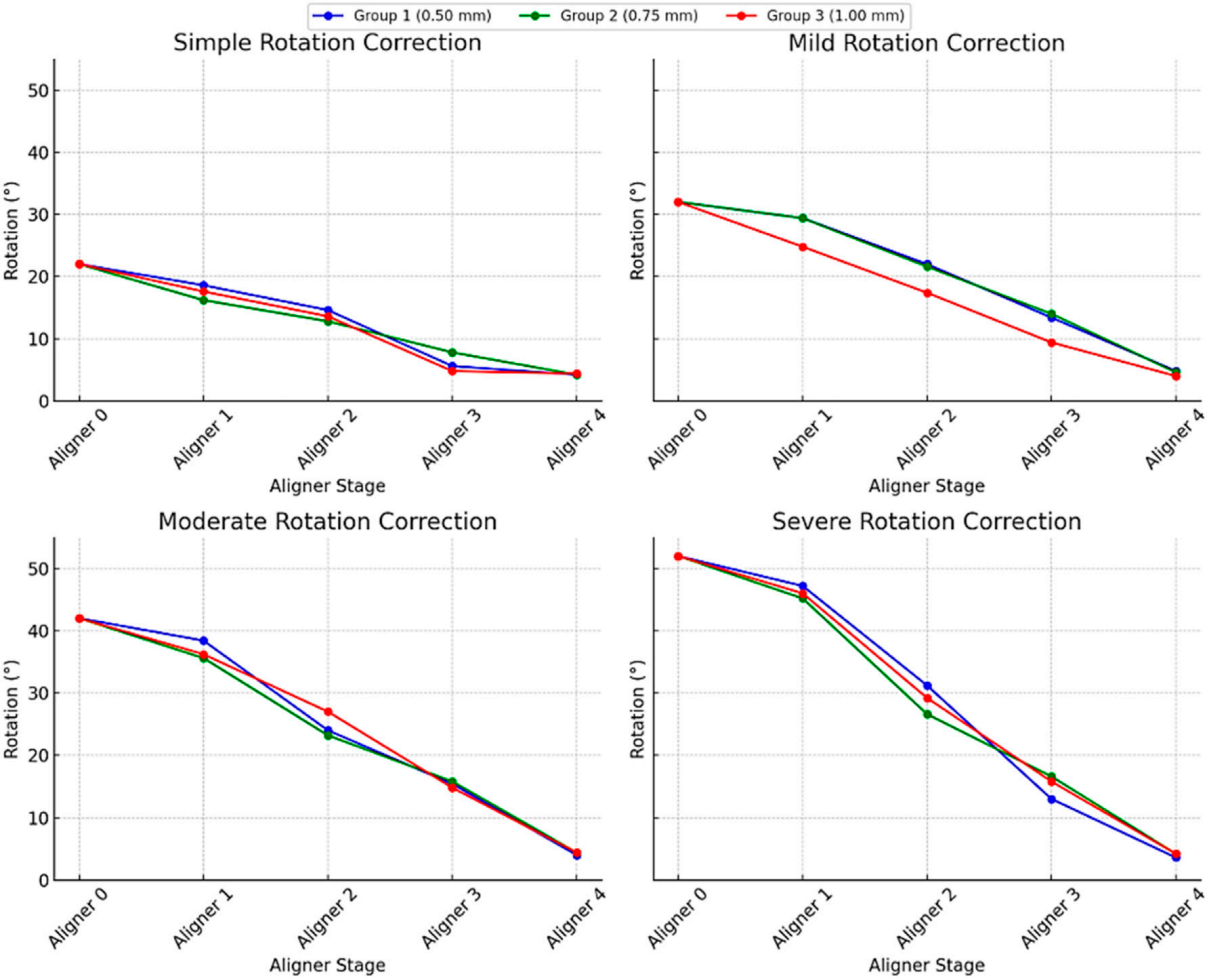
Degree of correction of a rotated maxillary right central incisor treated with 3D-printed orthodontic aligners of varying thicknesses across different initial rotation severities. In each severity graph, the lines correspond to different aligner thicknesses. The x-axis represents the sequential aligner number (treatment stage), while the y-axis shows the remaining degree of rotation (in degrees).

As presented in Table 1 and in Figure 6, by the final aligner stage (Aligner 4), all three thicknesses achieved nearly complete correction, with residual rotations narrowed to just 4°–5°. However, the pathways they took to get there differed slightly depending on aligner thickness and the severity of the initial rotation, hence the null hypothesis was rejected. Inter-group variability (see S1) in correction efficacy was low (SD = 0.5%–1.2% across aligner thicknesses), suggesting consistent clinical outcomes regardless of material thickness.

**TABLE 1:** The percentage correction of rotation of the maxillary central incisor per group.

	Aligner thickness		
Rotation Severity	Group 1 (0.50 mm)	Group 2 (0.75 mm)	Group 3 (1.00 mm)
Simple (22°)	80.91%	80.91%	80.00%
Mild (32°)	85.00%	85.63%	87.50%
Moderate (42°)	90.48%	89.52%	89.52%
Severe (52°)	93.08%	91.92%	91.92%

For the mildest cases (initial 22° rotation), the 0.50 mm and 1.00 mm aligners delivered larger early gains, reducing angular displacement to 14.6° and 13.6° respectively by Aligner 2, whereas the 0.75 mm aligner reached only 12.8° at the same point. Midway through treatment, the thinnest (0.50 mm) aligner made the most dramatic single‐stage jump, dropping to 5.6° by Aligner 3, while the 0.75 mm and 1.00 mm aligners trailed slightly behind at 7.8° and 4.8°. Despite these differences, all three converged at roughly 4.0°–4.4° residual rotation by Aligner 4 (p < 0.001, significant difference among aligners).

In mild and moderate rotations (initial 32° and 42°), a similar pattern emerged. For a 32° start, the 0.50 mm and 0.75 mm aligners moved the tooth down to about 22° by Aligner 2, leaving the 1.00 mm aligner behind at 17.4°. By mid‐treatment, the thinner aligners had reduced rotation by around 9°, whereas the thicker aligners gained 7.4°. In the 42° cases, the 1.00 mm aligner outpaced the others early on, dropping to 27.0° at Aligner 2 compared to 24.0° and 23.2° for the 0.50 mm and 0.75 mm aligners, yet ultimately all three reached the same 4.0°–4.4° degrees (*p* < 0.001, statistically significant across stages).

Even severe rotations (initial 52°) followed this convergence pattern. The thinnest aligner again made the biggest early move, lowering rotation to 31.2° by Aligner 2, versus 29.2° and 26.6° for the 1.00 mm and 0.75 mm aligners. The medium‐thickness aligner was then caught up by Aligner 3, and by the end, all aligners achieved residual rotations within a tight 3.6°–4.4° range (p < 0.001). In short, while the ultimate outcomes were equivalent, the 0.50 mm and 1.00 mm aligners produced faster early‐stage correction (*p* < 0.001, significant early improvement), and the 0.75 mm aligners delivered a steadier, more uniform progression.

Aligners achieved 80.0%–93.1% of planned rotational corrections, with greater relative correction efficiency observed in more severe initial rotations (91.9%–93.1% for severe) compared to simpler cases (80.0%–80.9%) (p < 0.001, significant difference among rotation severities).

## Discussion

Clear aligners have gained popularity as a modern alternative to traditional fixed braces, offering improved aesthetics and enhanced comfort for patients. Numerous numerical and experimental methods have been used to evaluate their effectiveness, including typodont-based simulations without sensors, which serve as reliable pre-clinical models for studying sequential aligner performance (Elshazly et al., 2021). In the current study, a novel electric wax-block typodont was utilized. Each tooth in this model is embedded in a heat-sensitive wax material that softens during a controlled heating phase, simulating the biomechanical properties of the periodontal ligament, and hardens during cooling. This cycle allows the forces from the aligner to move the teeth in a measurable and reproducible manner. The use of rigid guiding stents allows for rapid repositioning of the teeth, enabling multiple consistent trials and making this model a practical and dependable method for comparing different aligner variables, such as thickness or staging, before clinical use.

Among all tooth movements, rotating incisors remains particularly difficult, especially when treated with clear aligners instead of fixed braces (Bowman, 2020). This highlights the need to better understand and optimize factors that affect rotational control. One key factor influencing aligner performance is its thickness (Ghoraba et al., 2024), which directly affects the force exerted and, in turn, the degree of tooth movement (Elkholy et al., 2017; Elshazly et al., 2024b). Some treatment protocols intentionally vary thickness during different stages to manage force levels, similar to how fixed appliances function (Elkholy et al., 2017; Bucci et al., 2019; Jindal et al., 2020; Edelmann et al., 2020; Iliadi et al., 2019). However, the thermoforming process itself can unpredictably alter the intended material thickness (Elshazly et al., 2022b) and negatively affect the mechanical and physical properties of the aligner material (Ryu et al., 2018). To address this, advanced materials with enhanced properties have been explored to improve aligner performance (Elshazly et al., 2022a). Shape memory polymers (SMPs) combined with 3D printing (4D Aligners) offer a more precise, economical, and environmentally sustainable solution (Atta et al., 2023; Jindal et al., 2020; Sharif et al., 2024).

In this study, aligners were 3D-printed in a horizontal orientation. However, recent studies (McCarty et al., 2020; Camenisch et al., 2024) have shown that printing direction does not significantly influence the aligners’ mechanical behavior. All aligners underwent a 25-min UV curing process, following manufacturer guidelines. Previous work (Bleilöb et al., 2025) has confirmed that a 20-min cure is sufficient to ensure biocompatibility for thicknesses up to 6 mm. A 2-mm straight trimming line was also applied. Supporting previous findings (Elshazly et al., 2022b; Elshazly et al., 2024b; Elshazly et al., 2024c), this trimming design helped distribute stress more evenly and increased force delivery closer to the tooth’s center of resistance, enhancing movement control.

The experimental model effectively demonstrated the aligners’ capacity to rotate teeth. All three aligner thicknesses (0.50, 0.75, and 1.00 mm) effectively corrected varying degrees of incisor rotation, from mild to severe, but left a residual rotation of about 4°–5° rather than achieving the full 0°. Such an incomplete correction is consistent with previous reports (Koletsi et al., 2021) in clear aligner therapy, and may be attributed to factors including material elasticity, attachment design and application, aligner adaptability, and individual anatomical variability (Upadhyay and Arqub, 2022). Given the small magnitude of this residual rotation, its clinical relevance is likely limited, particularly when it falls within the range of acceptable occlusal and esthetic outcomes.

Early-stage differences were observed: thinner (0.50 mm) and thicker (1.00 mm) aligners corrected rotation more quickly at the beginning, while the medium-thickness (0.75 mm) aligner offered a steadier, more controlled correction. Ultimately, all groups reached similar final results, with no statistically significant differences in total rotation achieved. This contrasts with some earlier reports (Ghoraba et al., 2024; Ryu et al., 2018) suggesting that thicker aligners produce stronger and longer-lasting forces. However, it supports other studies (Elkholy et al., 2017; Bucci et al., 2019) indicating that thickness has minimal influence on clinical outcomes, particularly with 3D-printed aligners. This may be due to minor deviations between digitally planned and printed thicknesses caused by printing limitations (Edelmann et al., 2020).

Interestingly, this study showed that rotated incisors could be successfully corrected using aligners without attachments, challenging earlier recommendations and previous reports (Cortona et al., 2020; Jones et al., 2009; Gomez et al., 2015). These findings align with more recent research (Elshazly et al., 2024c; Kravitz et al., 2008; Simon et al., 2014) indicating that effective tooth movement can be achieved using strategies like high trimming lines and varied thicknesses instead of relying solely on attachments. Moreover, the material’s shape memory characteristics, previously documented (Atta et al., 2023; Lee et al., 2022), contribute to improved adaptability (Elshazly et al., 2022b; Koenig et al., 2022), prolonged force application, and the potential for greater incremental tooth movement per aligner (Sharif et al., 2024), which enhances the control of tooth movement.

The observed differences in early-stage correction among aligners of varying thicknesses can be attributed to their distinct biomechanical properties. Thinner aligners (0.50 mm) exhibit greater flexibility, allowing for rapid force application, which may facilitate quicker initial tooth movement. Conversely, thicker aligners (1.00 mm) provide increased stiffness, delivering higher forces that can lead to more substantial early-stage correction (Elshazly et al., 2024a; Elkholy et al., 2017). The intermediate 0.75 mm aligner likely provides more controlled, sustained forces, resulting in slower but steadier tooth movement. These observations align with fundamental biomechanical principles, where both force magnitude and duration influence the rate and predictability of tooth displacement (Upadhyay and Arqub, 2022).

Despite its usefulness, the typodont model has limitations. It does not replicate the full complexity of the oral environment, such as varying temperatures, saliva, or biting forces, all of which can influence aligner behavior. Additionally, precise virtual setup and 3D-printing workflows require significant expertise. Moreover, due to the equipment limitations in our laboratory, measuring tooth rotation was performed manually using a protractor instead of digital model superimposition. The use of a manual protractor for measurements may introduce minor measurement inaccuracies and limit the precision of rotational assessment. Future studies may use superimposition of digital scans and will include measuring the actual forces and torques applied by the aligners. Furthermore, clinical trials are also necessary to validate these findings under real-life conditions.

## Conclusion

Within the limitations of the current *in vitro* study, we could draw the following conclusions:

1. The electric typodont proved to be a reliable preclinical model for simulating tooth movement induced by orthodontic aligners.
2. 3D-printed aligners successfully corrected rotated anterior teeth across varying severities in the typodont model, even without the use of auxiliary attachments.
3. All aligner thicknesses achieved similar final correction but differed in timing: 0.50 mm and 1.00 mm aligners showed faster early-stage movement, while 0.75 mm aligners provided more gradual, consistent correction.

## Clinical implications

This study introduced a practical, standardized method for the preclinical assessment of clear aligners, providing valuable insights prior to clinical application. It also demonstrated that 3D-printed aligners can effectively correct anterior tooth rotations of varying severities without the need for attachments. While aligner thickness influenced the rate of derotation, it did not affect the final correction outcome, enabling clinicians to tailor treatment dynamics—such as rapid initial movement versus steady progression—to individual patient needs.

## Data Availability

The datasets presented in this study can be found in online repositories. The names of the repository/repositories and accession number(s) can be found below: https://data.mendeley.com/datasets/vnhjpv675p/1.

## Footnotes

1
https://savariadent.hu/en/product/savaria-dent-product-family/electrodont/electrodont
