# Supplementary material for: Preclinical evaluation of 3D-Printed orthodontic aligners using an electro-typodont model
Source: Front Bioeng Biotechnol. 2025 Nov 10;13:1650447. doi: 10.3389/fbioe.2025.1650447 (PMC12641186; doi:10.3389/fbioe.2025.1650447)
Supplement: Supplementary file 1 [file Table1.docx]

Supplement Tables

Table 1: Mean ± SD rotational values for different aligner stages (Aligner 0–4) in Group 1 (0.50 mm). All rotation levels (simple, mild, and moderate) showed a progressive reduction throughout treatment, and the differences between aligners were statistically significant (*p* < 0.001).

|  | **Group 1 (0.50 mm)** | | | | |  |
| --- | --- | --- | --- | --- | --- | --- |
| Rotation | Aligner 0 | Aligner 1 | Aligner 2 | Aligner 3 | Aligner 4 | p-value |
|  | Mean ± SD | Mean ± SD | Mean ± SD | Mean ± SD | Mean ± SD |  |
| simple | 22 ± 0 | 18.6 ± .548 | 14.6 ± .548 | 5.6 ± .548 | 4.2 ± .447 | <.001* |
| mild | 32 ± 0 | 29.4 ± .548 | 22.0 ± 1.871 | 13.4 ± .548 | 4.8 ± .447 | <.001* |
| moderate | 42 ± 0 | 38.4 ± .548 | 24.0 ± 1.00 | 15.4 ± .548 | 4.0 ± .707 | <.001* |
| severe | 52 ± 0 | 47.2 ± .837 | 31.2 ± .837 | 13.0 ± .707 | 3.6 ± .548 | <.001* |

Table 2: Mean ± SD rotational values for different aligner stages (Aligner 0–4) in Group 2 (0.75 mm). All rotation levels (simple, mild, and moderate) showed a progressive reduction throughout treatment, and the differences between aligners were statistically significant (*p* < 0.001).

|  | **Group 2 (0.75 mm)** | | | | |  |
| --- | --- | --- | --- | --- | --- | --- |
| Rotation | Aligner 0 | Aligner 1 | Aligner 2 | Aligner 3 | Aligner 4 | p-value |
|  | Mean ± SD | Mean ± SD | Mean ± SD | Mean ± SD | Mean ± SD |  |
| simple | 22 ± 0 | 16.2 ± .447 | 12.8 ± 1.64 | 7.8 ± .837 | 4.2 ± .447 | <.001* |
| mild | 32 ± 0 | 29.4 ± .548 | 21.6 ± 1.52 | 14.0 ± .707 | 4.6 ± .548 | <.001* |
| moderate | 42 ± 0 | 35.6 ± .548 | 23.2 ± 2.68 | 15.8 ± .447 | 4.4 ± .548 | <.001* |
| severe | 52 ± 0 | 45.2 ± .837 | 26.6 ± .548 | 16.6 ± .548 | 4.2 ± .447 | <.001* |

Table 3: Mean ± SD rotational values for different aligner stages (Aligner 0–4) in Group 3 (1.00 mm). All rotation levels (simple, mild, and moderate) showed a progressive reduction throughout treatment, and the differences between aligners were statistically significant (*p* < 0.001).

|  | Group 3 (1.00 mm) | | | | |  |
| --- | --- | --- | --- | --- | --- | --- |
| Rotation | Aligner 0 | Aligner 1 | Aligner 2 | Aligner 3 | Aligner 4 | p-value |
|  | Mean ± SD | Mean ± SD | Mean ± SD | Mean ± SD | Mean ± SD |  |
| simple | 22 ± 0 | 17.6 ± .548 | 13.6 ± .548 | 4.8 ± .447 | 4.4 ± .548 | <.001* |
| mild | 32 ± 0 | 24.8 ± 1.30 | 17.4 ± 2.61 | 9.4 ± 1.52 | 4.0 ± .707 | <.001* |
| moderate | 42 ± 0 | 36.2 ± .837 | 27.0 ± 1.00 | 14.8 ± .837 | 4.4 ± .548 | <.001* |
| severe | 52 ± 0 | 46.0 ± 1.00 | 29.2 ± .837 | 15.8 ± .837 | 4.2 ± .447 | <.001* |
